# A Case Series of Diarrheal Diseases Associated with *Yersinia frederiksenii*

**DOI:** 10.3390/idr13020051

**Published:** 2021-06-13

**Authors:** Eugene Y. H. Yeung

**Affiliations:** Department of Medical Microbiology, The Ottawa Hospital General Campus, The University of Ottawa, Ottawa, ON K1H 8L6, Canada; eugeney@doctors.org.uk

**Keywords:** *Yersinia frederiksenii*, *Yersinia enterocolitica*, yersiniosis, diarrhea, microbial sensitivity tests, stool culture, sulfamethoxazole-trimethoprim, gastroenteritis

## Abstract

To date, *Yersinia pestis*, *Yersinia enterocolitica*, and *Yersinia pseudotuberculosis* are the three *Yersinia* species generally agreed to be pathogenic in humans. However, there are a limited number of studies that suggest some of the “non-pathogenic” *Yersinia* species may also cause infections. For instance, *Yersinia frederiksenii* used to be known as an atypical *Y. enterocolitica* strain until rhamnose biochemical testing was found to distinguish between these two species in the 1980s. From our regional microbiology laboratory records of 18 hospitals in Eastern Ontario, Canada from 1 May 2018 to 1 May 2021, we identified two patients with *Y. frederiksenii* isolates in their stool cultures, along with their clinical presentation and antimicrobial management. Both patients presented with diarrhea, abdominal pain, and vomiting for 5 days before presentation to hospital. One patient received a 10-day course of sulfamethoxazole-trimethoprim; his *Y. frederiksenii* isolate was shown to be susceptible to amoxicillin-clavulanate, ceftriaxone, ciprofloxacin, and sulfamethoxazole-trimethoprim, but resistant to ampicillin. The other patient was sent home from the emergency department and did not require antimicrobials and additional medical attention. This case series illustrated that diarrheal disease could be associated with *Y. frederiksenii*; the need for antimicrobial treatment should be determined on a case-by-case basis.

## 1. Introduction

Yersiniosis is a foodborne infectious disease that causes fever, abdominal pain, and often bloody diarrhea, which generally develop 4 to 7 days after exposure and last 1 to 3 weeks [1,2]. Yersiniosis is usually acquired by the ingestion and handling of contaminated food, especially raw or undercooked pork, contaminated milk, and untreated water [2,3]. *Yersinia enterocolitica*, and less often *Yersinia pseudotuberculosis*, are gram-negative bacterial species that cause human intestinal yersiniosis [2]. Pigs are the major animal reservoir of these species, although rabbits, sheep, cattle, horses, dogs, and cats can also carry strains that infect humans [2]. For hospital inpatients, the Centers for Disease Control and Prevention (CDC) recommends contact precautions for diapered or incontinent persons for the duration of illness or to control outbreaks [4]. However, yersiniosis is an uncommon disease in Canada, accounting for 288 cases (rate of 2 per 100,000), 15 hospitalizations, and no deaths in Ontario, Canada in 2019 [1]. Yersiniosis is generally a self-limiting disease that requires mainly supportive therapy; antibiotics are reserved for complicated infections, such as sepsis [2,3]. In a study of 67 patients with an *Y. enterocolitica* infection, antimicrobial treatment was not associated with a decreased duration of illness (18 and 21 days with and without antimicrobials, respectively) [5]. Therefore, antimicrobial susceptibility testing for *Yersinia* species is not always warranted.

To date, *Yersinia pestis*, *Y. enterocolitica*, and *Y. pseudotuberculosis* are the three *Yersinia* species generally agreed to be pathogenic in humans. They share a virulence plasmid (called pCD1 in *Y. pestis* and pYV in *Y. enterocolitica* and *Y. pseudotuberculosis*) and a chromosomal high-pathogenicity island (HPI), whereas the rest of the species in the *Yersinia* genus lack these virulence plasmid and chromosomal proteins [6]. Although *Y. pestis* does not generally cause gastrointestinal symptoms, it is the etiologic pathogen that causes life-threatening plague, which is usually transmitted to humans by the bite of an infected rat flea vector [3,6]. *Y. pestis* infection can also be transmitted through contact with a fluid or tissue from an infected animal and inhaling droplets from patients with pulmonary infection [3,4]. The CDC recommends droplet precaution for the source patient with pneumonic plague until 48 h after effective antibiotic therapy [4].

There were a limited number of published studies that suggested the “non-pathogenic” *Yersinia* species might also cause symptomatic infections in humans. For instance, in a study of 38 patients with non-pathogenic *Yersinia* species isolates (12 *Yersinia intermedia*, 7 *Yersinia frederiksenii*, 3 *Yersinia kristensenii*, and 16 unclassified *Yersinia* isolates), 10 patients had diarrhea alone [7]. The diarrheal symptoms persisted for less than a week in 21% and more than a month in 54% of the cases. Other contributory factors to their symptoms included prior use of antimicrobials and concurrent use of corticosteroids and proton pump inhibitors.

In our microbiology laboratory in Eastern Ontario Regional Laboratory Association (EORLA), we retrospectively audited stool cultures in the eighteen affiliated hospitals. After reviewing all of the patients with *Yersinia* isolates in their stool cultures from 1 May 2018 to 1 May 2021 (3-year inclusive), we identified eleven patients—two with *Y. frederiksenii* and nine with *Y. enterocolitica* isolates. The current quality improvement project detailed the clinical presentations of patients with *Y. frederiksenii* isolates in their stool cultures, which are generally known as non-pathogens [6]. The current project aimed to determine how antimicrobial susceptibility testing and treatment impacted patients’ clinical management.

## 2. Materials and Methods

The current quality improvement project followed the guidelines and standards given by the Ottawa Health Science Network Research Ethics Board. Eighteen hospitals in Eastern Ontario (the Almonte General Hospital, Arnprior Regional Health, Carleton Place and District Memorial Hospital, Children’s Hospital of Eastern Ontario, Cornwall Community Hospital, Deep River and District Hospital, Glengarry Memorial Hospital, Hawkesbury District General Hospital, Kemptville District Hospital, Montfort Hospital, Pembroke Regional Hospital, Queensway Carleton Hospital, Renfrew Victoria Hospital, St. Francis Memorial Hospital, Ottawa General Hospital, Ottawa Civic Hospital, Ottawa Riverside Hospital, and Winchester District Memorial Hospital) submitted patients’ stool samples to the EORLA regional microbiology laboratory for testing. The laboratory used the Cerner Millennium software (Version 2013.04.1.34; Kansas City, MO, USA) to store the patients’ laboratory data. This software generated reports that included all of the patients with *Yersinia* species isolated in their stool cultures in a three-year period (1 May 2018 to 1 May 2021). To reduce the risk of false positive and negative results, the microbiology laboratory would only perform cultures on the patients’ stool specimens if they were transported to with Cary Blair Transport Medium, which had been internally validated for use.

The stool specimens were cultured on MacConkey agar plates at 35 °C for 24 h in an oxygen incubator, where colonies of non-lactose fermenting microorganisms (such as *Salmonella*, *Shigella*, and *Yersinia* species) would be suggestive of stool pathogens and thereby undergo further testing. To improve the yield of *Yersinia* species, the MacConkey plates were left at room temperature for an additional 24 h, where pinpoint colonies would be suggestive of stool pathogens and thereby undergo further testing. To improve the yield of *Salmonella* and *Shigella* species, the stool specimens were cultured on Hektoen selective agar plates at 35 °C for 24 h in an oxygen incubator. To improve the yield of *Escherichia coli* O157, the stool specimens were also cultured on sorbitol MacConkey selective agar plates 35 °C for 24 h in an oxygen incubator. To improve the yield of *Campylobacter* species, the stool specimens were also cultured on *Campylobacter* selective agar plates at 42 °C for 48 h in sealed jars with CamyGen sachets. The microorganisms isolated from the above agar plates were further identified using Bruker matrix-assisted laser desorption ionization-time of flight mass spectrometry (MALDI-TOF MS). Each microorganism was identified to species level when the MALDI-TOF score was ≥2.0. The antimicrobial susceptibility results were based on Clinical and Laboratory Standards Institute (CLSI) Kirby–Bauer inhibition zone breakpoints. However, antimicrobial susceptibility testing was not routinely performed on *Yersinia* isolates in stools unless specifically requested by the clinicians, as there is a lack of evidence to support the benefits of antimicrobial therapy [5].

Each patient’s electronic health record was retrospectively reviewed using the software Epic Hyperspace (Version November 2018, Verona, MI, USA). Their demographics, clinical history, and therapeutic records were recorded on a separate spreadsheet.

## 3. Results

Patient A was a 66-year-old male with a history of asthma, chronic obstructive pulmonary disease, depression, gastroesophageal reflux disease, myocardial infarction, pulmonary fibrosis, and substance use disorder, who resided in a homeless shelter. He had *Clostridioides difficile* diarrhea diagnosed using a *C. difficile* toxin B polymerase chain reaction (PCR) assay about a month prior to his current hospital admission and was treated with 21 days of oral vancomycin and 10 days of intravenous metronidazole. He presented to hospital again with vomiting, loose stool, and abdominal pain for 5 days when he was not on antimicrobials. He was afebrile and hemodynamically stable. His bloodwork results on arrival were as follows: white blood cell count 10.3 × 10^9^/L; platelet 362 × 10^9^/L; sodium 131 mmol/L; creatinine 72 umol/L; potassium 3.6 mmol/L; and lactate 2.4 mmol/L. Computed tomography images of his abdomen and pelvis revealed dilated small and large bowels and increased intraluminal fluid, suggestive of enterocolitis. His stool sample was processed in the microbiology laboratory, which reported negative *C. difficile* PCR but growth of *Y. frederiksenii* on the culture plates. No other stool pathogens were identified. At that time, the patient had been having ongoing diarrhea in hospital at least three times a day for four days. The infectious diseases team was consulted and requested antimicrobial susceptibility testing. The patient was placed on contact precaution because of his acute gastroenteritis symptoms. In view of the patient’s ongoing symptoms, the infectious diseases team opted to start the patient on one sulfamethoxazole-trimethoprim double strength tablet (800 mg/160 mg per tablet) twice a day for 10 days. Antimicrobial susceptibility testing was performed on his *Y. frederiksenii* isolate with Kirby–Bauer discs (Table 1). For the purpose of antimicrobial stewardship [8], our laboratory cascade reported the isolate’s susceptibility to amoxicillin-clavulanate, ceftriaxone, ciprofloxacin, and sulfamethoxazole-trimethoprim, and resistance to ampicillin only. No change was made to Patient A’s antimicrobial therapy as his *Y. frederiksenii* isolate was susceptible to sulfamethoxazole-trimethoprim. On day five of the sulfamethoxazole-trimethoprim therapy, the patient was discharged home in a stable condition. His blood cultures, *Helicobacter pylori* IgG, stool ova, and parasite testing results were found to be negative later. The patient re-visited the emergency department 4 days after hospital discharge because of mood and housing issues. During the visit, the patient denied having diarrhea, suggesting the sulfamethoxazole-trimethoprim therapy was effective in curing his diarrheal illness.

Patient B was a 35-year-old female with a history of anxiety and gastroesophageal reflux disease (*H. pylori* IgG non-reactive) who presented with diarrhea, abdominal pain, nausea, and vomiting for 5 days. She was afebrile and hemodynamically stable. No bloodwork and radiological imaging were ordered. She was given one dose of 50 mg dimenhydrinate and sent home from the emergency department. Her stool ova and parasite testing were negative, but a culture showed *Y. frederiksenii* isolates. No other stool pathogens were identified. The emergency department staff gave the patient a phone call 4 days after her visit and disclosed the microbiological diagnosis. However, she required no antimicrobials or further medical attention. Antimicrobial susceptibility testing was not performed on her *Y. frederiksenii* isolates.

## 4. Discussion

*Y. frederiksenii* used to be known as an atypical *Y. enterocolitica* strain until rhamnose biochemical testing was found to distinguish between these two species in the 1980s [9]. *Y. frederiksenii* could be differentiated from *Y. pseudotuberculosis* by showing positive reactions for ornithine decarboxylase, indole, sucrose, sorbose, sorbitol, inositol, and Voges-Proskauer [9]. As evidenced in this case series, *Y. frederiksenii* could also cause diarrheal diseases such as *Y. enterocolitica* and *Y. pseudotuberculosis*, the two etiologic agents for yersiniosis. *Y. frederiksenii* could be contributory to the 5 days of abdominal pain and vomiting these two patients experienced. As evidenced in Patient A, untreated *Y. frederiksenii* might lead to complications such as enterocolitis and a high lactate level.

Nevertheless, the presence of *Y. frederiksenii* in stools does not always warrant the use of antimicrobials. Patient B required no antimicrobials or further medical attention, despite being symptomatic at initial presentation with *Y. frederiksenii* found in the stool culture. This finding was consistent with a study of 38 patients with non-pathogenic *Yersinia* species isolates (12 *Y. intermedia*, 7 *Y. frederiksenii*, 3 *Y. kristensenii*, and 16 unclassified *Yersinia* isolates), where only five patients required antimicrobial treatment; two patients received ciprofloxacin that resulted in symptoms for more than 1 month; one patient received erythromycin that resulted in symptoms for less than a week; and two patients received metronidazole and had no follow-ups. [7]. However, it was unclear which *Yersinia* species these five patients contracted.

The fact that *Yersinia* diarrheal diseases may self-resolve justifies why our microbiology laboratory does not routinely perform antimicrobial susceptibility testing on *Yersinia* isolates in stool, unless requested by clinicians. If antimicrobial susceptibility tests were performed for all patients, their clinicians might be misled to prescribe antimicrobials even if they are not needed. Patient A’s *Y. frederiksenii* isolates showed susceptibility to amoxicillin-clavulanate, ceftriaxone, ciprofloxacin, and sulfamethoxazole-trimethoprim, but resistance to ampicillin. The findings were consistent with a study of 38 *Y. frederiksenii* strains collected in France, Germany, Sweden, Switzerland, and Norway, where 36 strains were susceptible to amoxicillin-clavulanate, all of the strains were susceptible to ceftriaxone, ciprofloxacin, and sulfamethoxazole-trimethoprim, and all of the strains were resistant to ampicillin or amoxicillin (as per the CLSI minimum inhibitory concentration breakpoints) [10]. However, the need for antimicrobial treatment should be determined on a case-by-case basis.

Patient A was put on contact precaution due to his gastroenteritis symptoms. There is currently a lack of evidence to suggest that *Y. frederiksenii* could be transmitted through direct person-to-person contact. An infection control study was conducted on a 24-year-old female hospital resident physician, who was diagnosed to have a gastrointestinal infection with *Y. frederiksenii* in 1993 [11]. Her nine co-residents were screened for fecal carriage of *Y. frederiksenii*—three of them were tested positive but none of them had symptoms. Despite carrying out surveillance studies on the symptomatic resident physician’s unpasteurized milk and hospital holding tanks, the infection control team failed to identify the source of her *Y. frederiksenii* infection.

The current study added to the debate as to whether the “non-pathogenic” *Yersinia* species causes symptomatic infections in humans. Despite our best efforts, our regional laboratory captured only two cases of “non-pathogenic” *Yersinia* species isolated from stool cultures in a 36-month period. The rare incidence rate of Yersiniosis (2 per 100,000 in Ontario [1]) led to the small sample size, a major limitation of the current study. Moreover, our laboratory would only process cultures on the stool samples collected in Cary Blair Transport Medium. It is possible that there were more patients with yersiniosis whose stool samples were rejected in the laboratory due to the incorrect containers used. The current case patients did not undergo viral gastroenteritis testing; a positive result would suggest co-infection or an alternative diagnosis. Nevertheless, viral gastroenteritis tends to be a self-limiting disease in immunocompetent adults that do not always warrant laboratory investigations in clinical practice [12]. Unlike previous studies on non-pathogenic *Yersinia* species [7,10], the current quality improvement project was specific to *Y. frederiksenii*. Despite the small sample size, the current case series demonstrated that *Y. frederiksenii* isolates in stool did not always require antimicrobial treatment. The antimicrobial susceptibility profile of *Y. frederiksenii* in our laboratory in Eastern Ontario, Canada, was consistent with the published data in Europe [10].

Future quality improvement projects should try to capture more cases of *Yersinia* isolates in stool. This could be performed by a continual auditing of the microbiology laboratory stool culture results, along with patients’ clinical histories and therapeutic records. The laboratory may consider the use of selective agar plates specific for *Yersinia* species to further improve the positivity rate [13,14,15]. Better education and communication with the clinicians may improve the use of correct containers to transport stool for culturing.

## 5. Conclusions

The results were inconclusive on whether the *Y. frederiksenii* isolates in stool were pathogenic isolates that required antimicrobial treatment and isolation precaution. If antimicrobials are needed, *Y. frederiksenii* isolates have been shown to be susceptible to amoxicillin-clavulanate, ceftriaxone, ciprofloxacin, and sulfamethoxazole-trimethoprim, but resistant to ampicillin. More data is needed to help clinicians to determine the best management of *Y. frederiksenii* isolates in stools.

## Figures and Tables

**Table 1:** Antimicrobial susceptibility of Patient A’s *Y. frederiksenii* isolate in stool culture (with Kirby–Bauer zone of inhibition diameter in millimeters) *.

Gentamicin	Susceptible	27 mm
Tobramycin	Susceptible	28 mm
Amikacin	Susceptible	25 mm
Amoxicillin-clavulanate	Susceptible	18 mm
Ampicillin	Resistant	7 mm
Cefazolin	Resistant	14 mm
Ceftriaxone	Susceptible	32 mm
Ciprofloxacin	Susceptible	37 mm
Ertapenem	Susceptible	38 mm
Meropenem	Susceptible	35 mm
Nitrofurantoin	Susceptible	21 mm
Piperacillin-tazobactam	Susceptible	30 mm
Sulfamethoxazole-trimethoprim	Susceptible	31 mm
Ceftazidime	Susceptible	29 mm
Cefpodoxime	Intermediate	20 mm

* The antimicrobial susceptibility results were interpreted using the Clinical and Laboratory Standards Institute (CLSI) breakpoints.

## Data Availability

The raw data presented in this study are available on request from the corresponding author. The data are not publicly available due to patient confidentiality.
